# Supplementary material for: Comparative multiomics analysis of cell physiological state after culture in a basket bioreactor
Source: Sci Rep. 2022 Nov 23;12:20161. doi: 10.1038/s41598-022-24687-4 (PMC9686226; doi:10.1038/s41598-022-24687-4)
Supplement: Supplementary file 1 — Supplementary Information 1. [file 41598_2022_24687_MOESM1_ESM.zip › raw data/Transcriptomic raw data/6.Enrichment/1.GO/TE1vsTC1/UP/TE1vsTC1.up.GOmf_DAG.pdf]

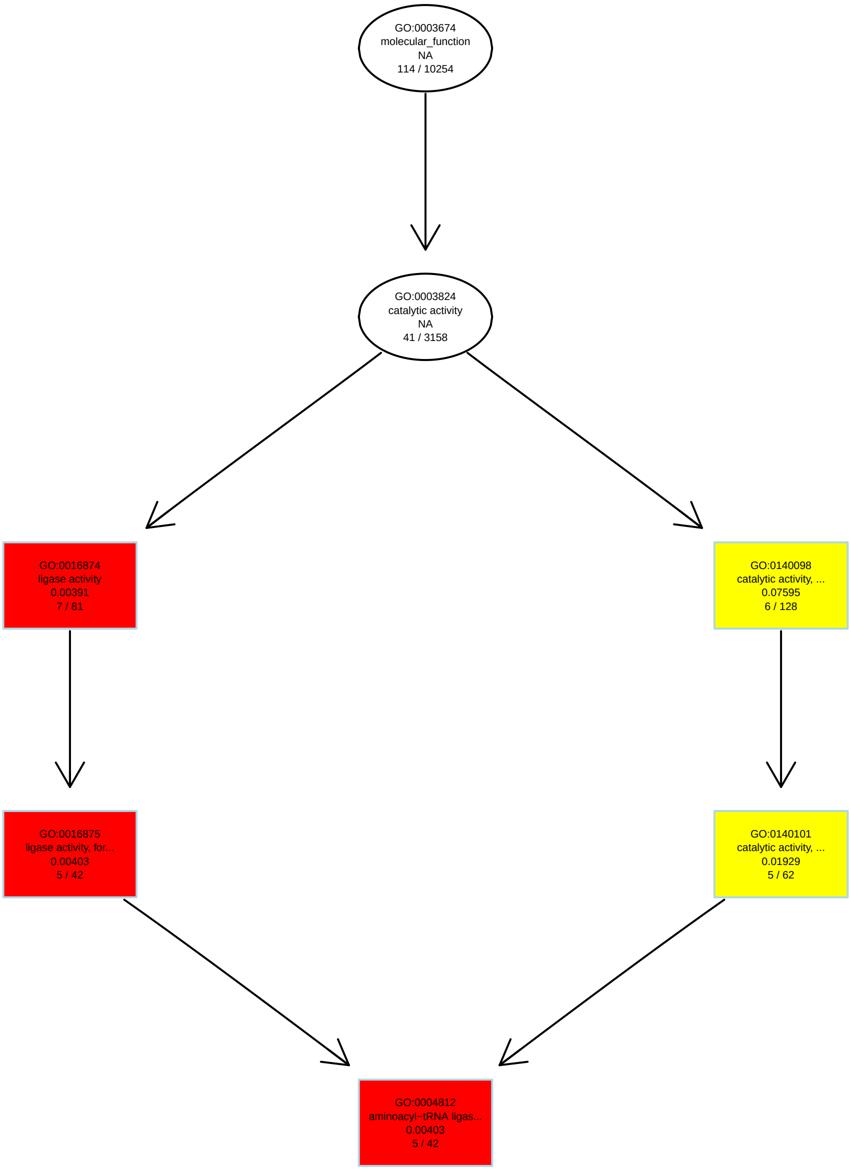
GO:0003674
molecular_function
NA
114 / 10254
GO:0003824
catalytic activity
NA
41 / 3158
GO:0016874
ligase activity
0.00391
7 / 81
GO:0140098
catalytic activity, ...
0.07595
6 / 128
GO:0016875
ligase activity, for...
0.00403
5 / 42
GO:0140101
catalytic activity, ...
0.01929
5 / 62
GO:0004812
aminoacyl−tRNA ligas...
0.00403
5 / 42
